# Characterization of hypermetabolic lymph nodes after SARS-CoV-2 vaccination using PET-CT derived node-RADS, in patients with melanoma

**DOI:** 10.1038/s41598-023-44215-2

**Published:** 2023-10-26

**Authors:** Antonio G. Gennari, Alexia Rossi, Thomas Sartoretti, Alexander Maurer, Stephan Skawran, Valerie Treyer, Elisabeth Sartoretti, Alessandra Curioni-Fontecedro, Moritz Schwyzer, Stephan Waelti, Martin W. Huellner, Michael Messerli

**Affiliations:** 1 Department of Nuclear Medicine, University Hospital Zurich, Rämistrasse 100, 8091 Zurich, Switzerland; 2 University of Zurich, Zurich, Switzerland; 3 Department of Medical Oncology and Hematology, University Hospital of Zurich, Zurich, Switzerland; 4 Department of Radiology and Nuclear Medicine, Children’s Hospital of Eastern Switzerland, St. Gallen, Switzerland

**Keywords:** Skin cancer, Diagnostic markers, Infectious-disease diagnostics

## Abstract

This study aimed to evaluate the diagnostic accuracy of Node Reporting and Data System (Node-RADS) in discriminating between normal, reactive, and metastatic axillary LNs in patients with melanoma who underwent SARS-CoV-2 vaccination. Patients with proven melanoma who underwent a 2-[^18^F]-fluoro-2-deoxy-D-glucose positron emission tomography/computed tomography (2-[^18^F]-FDG PET/CT) between February and April 2021 were included in this retrospective study. Primary melanoma site, vaccination status, injection site, and 2-[^18^F]-FDG PET/CT were used to classify axillary LNs into normal, inflammatory, and metastatic (combined classification). An adapted Node-RADS classification (A-Node-RADS) was generated based on LN anatomical characteristics on low-dose CT images and compared to the combined classification. 108 patients were included in the study (54 vaccinated). HALNs were detected in 42 patients (32.8%), of whom 97.6% were vaccinated. 172 LNs were classified as normal, 30 as inflammatory, and 14 as metastatic using the combined classification. 152, 22, 29, 12, and 1 LNs were classified A-Node-RADS 1, 2, 3, 4, and 5, respectively. Hence, 174, 29, and 13 LNs were deemed benign, equivocal, and metastatic. The concordance between the classifications was very good (Cohen’s *k*: 0.91, CI 0.86–0.95; *p*-value < 0.0001). A-Node-RADS can assist the classification of axillary LNs in melanoma patients who underwent 2-[^18^F]-FDG PET/CT and SARS-CoV-2 vaccination.

## Introduction

Since late 2019 the coronavirus disease (COVID-19) pandemic has deeply threatened global health. Up to now, almost 677 million people have been infected by the severe acute respiratory syndrome coronavirus 2 (SARS-CoV-2), worldwide^1^. Among them, roughly 6.9 million people died, mainly due to COVID-19 pulmonary complications. To limit the pandemic’s health, social, and economic impact an unprecedented international effort led to the rapid development of prophylactic vaccines, which proved to reduce SARS-CoV-2 infection and the number of COVID-19 severe/critical cases^2–4^.

However, following the introduction of the vaccines, reports of enlarged axillary lymph nodes (LNs) ipsilateral to the injection site, have been bourgeoning in literature^5–7^. In addition, several studies described metabolically active axillary LNs (HALNs) at 2-[^18^F]-fluoro-2-deoxy-D-glucose positron emission tomography/computed tomography (2-[^18^F]-FDG PET/CT), after vaccination^8,9^. Metabolically active or enlarged axillary LNs represent a diagnostic dilemma, particularly in patients with melanoma^9^ or breast cancer^5^ who were injected with the SARS-CoV-2 vaccine. To date, most of the studies have focused their attention on HALN frequency and dimension, whilst studies on anatomical criteria trying to characterize them are scarce^10–13^.

The Node Reporting and Data System (Node-RADS) has been recently released, aiming to standardize the LN reporting system^14^. Node-RADS provides a morphologic-based suspicion scale to predict LN involvement by malignancy. However, differentiating reactive from metastatic LNs relying solely on anatomical imaging is challenging. In addition, the benefits of adopting Node-RADS in cancer patients who underwent SARS-CoV-2 vaccination are unclear. Therefore, the aim of the present study was to evaluate the concordance between an adapted version of the Node-RADS based on unenhanced CT images, henceforth called A-Node-RADS, and clinical/2-[^18^F]-FDG PET findings in the characterization of axillary LNs in patients with melanoma, accounting for the vaccination status.

## Results

### Patient demographics

**Figure 1:**
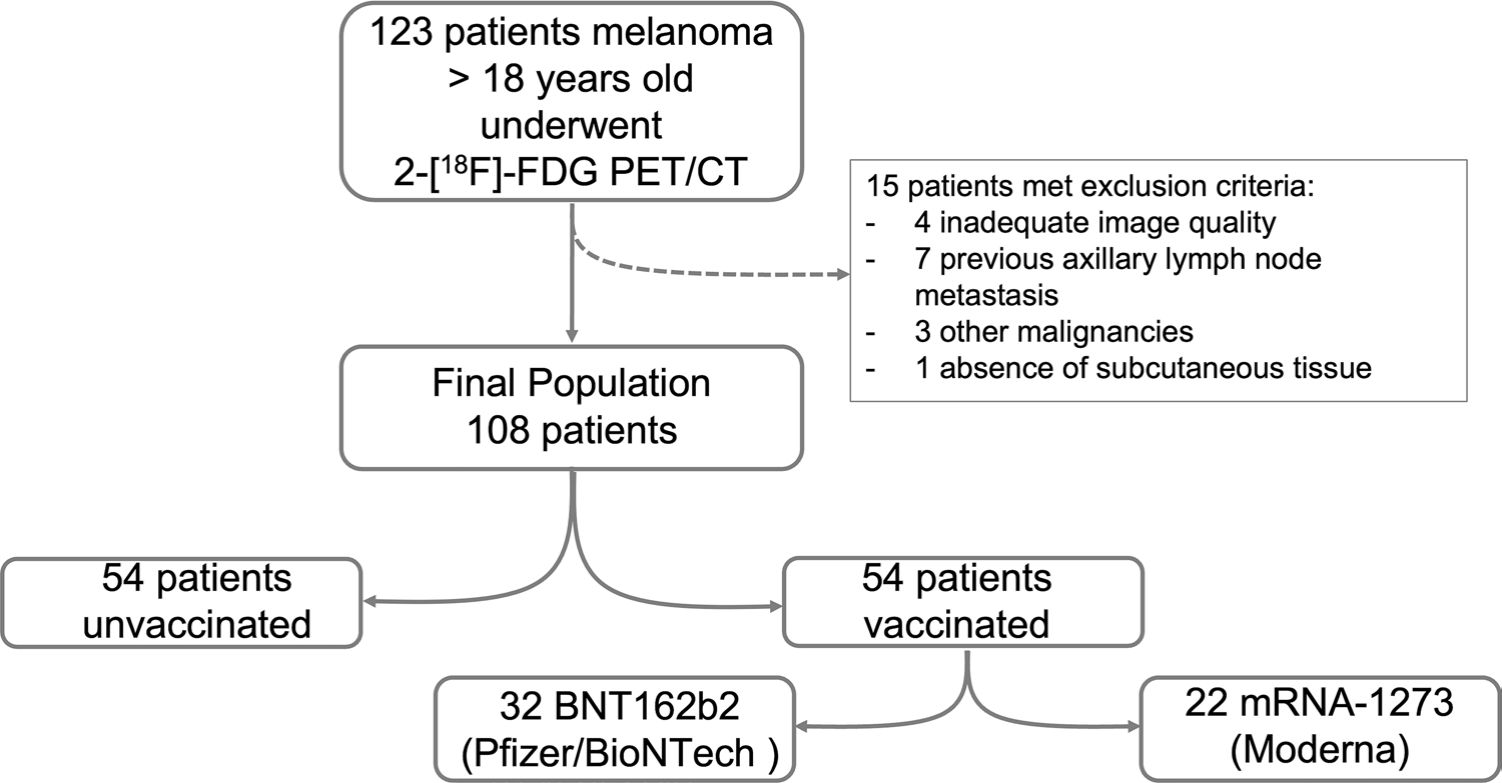
Flow diagram of the study group.

Hundred and twenty-three patients were considered eligible for this study, of which 15 were excluded from further analysis due to exclusion criteria. Thus, the final population of our study was 108 patients (Fig. 1). Patient demographics, vaccination status, type of vaccine injected, number of injections, previous SARS-CoV-2 infection, as well as primary melanoma site are reported in Table 1.

**Table 1:** Patient demographics.

	Vaccinated (n = 54)	Unvaccinated (n = 54)	p-value
Age, years	68.7 (13.9)	66.6 (14.5)	1.0
Sex, m	39 (72.2%)	37 (68.5%)	1.0
Vaccination			
Pfizer/BioNTech	32 (59.3%)		
Moderna Biontech	22 (40.7%)		
Vaccination site			
Left arm	45 (83.3%)		
Right arm	9 (16.7%)		
Number of injections			
Single	19 (35.2%)		
Double	35 (64.8%)		
Vaccine injection to 2-[^18^F]-FDG PET/CT scan, days			
Single injection	14.8 (8.0)		
Double injection	23.1 (12.7)		
Previous SARS-CoV-2 infection	2* (3.7%)	4 (7.4%)	1.0
Primary melanoma site			0.7
Head	23 (42.5%)	16 (29.6%)	0.55
Left arm	5 (9.3%)	4 (7.4%)	1.0
Right arm	2 (3.7%)	7 (13.0%)	0.81
Lower abdomen/genitalia	3 (5.6%)	1 (1.9%)	1.0
Back	7 (13.0%)	14 (25.9%)	0.98
Leg	10 (18.5%)	12 (22.2%)	1.0
Unknown	4 (7.4%)	0 (0%)	0.96

Data are expressed as mean (SD), absolute or relative values, as well as absolute or relative percentages.

*y* years, *SD* standard deviation, *SARS-CoV-2* severe acute respiratory syndrome coronavirus 2; *2-[*^*18*^*F]-FDG PET/CT* 2-[^18^F]-fluoro-2-deoxy-D-glucose positron emission tomography computed tomography.

*Both patients received Moderna Biontech.

Moderna was the most common vaccine injected in patients receiving a single dose of vaccine (63.2%; mean time between vaccination and 2-[^18^F]-FDG PET/CT scan: 14.6 days, range: 3–27 days), while Pfizer/BioNTech was more frequently used in those who were injected twice (71.4%; mean time between vaccination and 2-[^18^F]-FDG PET/CT scan: 21.5 days, range: 0–47 days). Age neither differed between vaccinated and unvaccinated patients (*p-*value = 0.59) nor between different vaccine cohorts (mean age_BNT162b2_: 69.9 years old, SD: 12.9, M = 24; mean age_mRNA-1273_: 67.3 years old, SD: 16.8, M = 15; *p-*value = 1). The left arm was the most common injection site (83.3% of vaccinated patients). Patients who received a second vaccine injection had longer latency to the 2-[^18^F]-FDG PET/CT compared to those who received a single dose (mean_single injection_: 14.8 days, SD: 8.0; mean_double injection_: 18.7 days, SD: 12.7; *p-*value = 0.013).

### LN 2-[^18^F]-FDG PET characteristics

**Figure 2:**
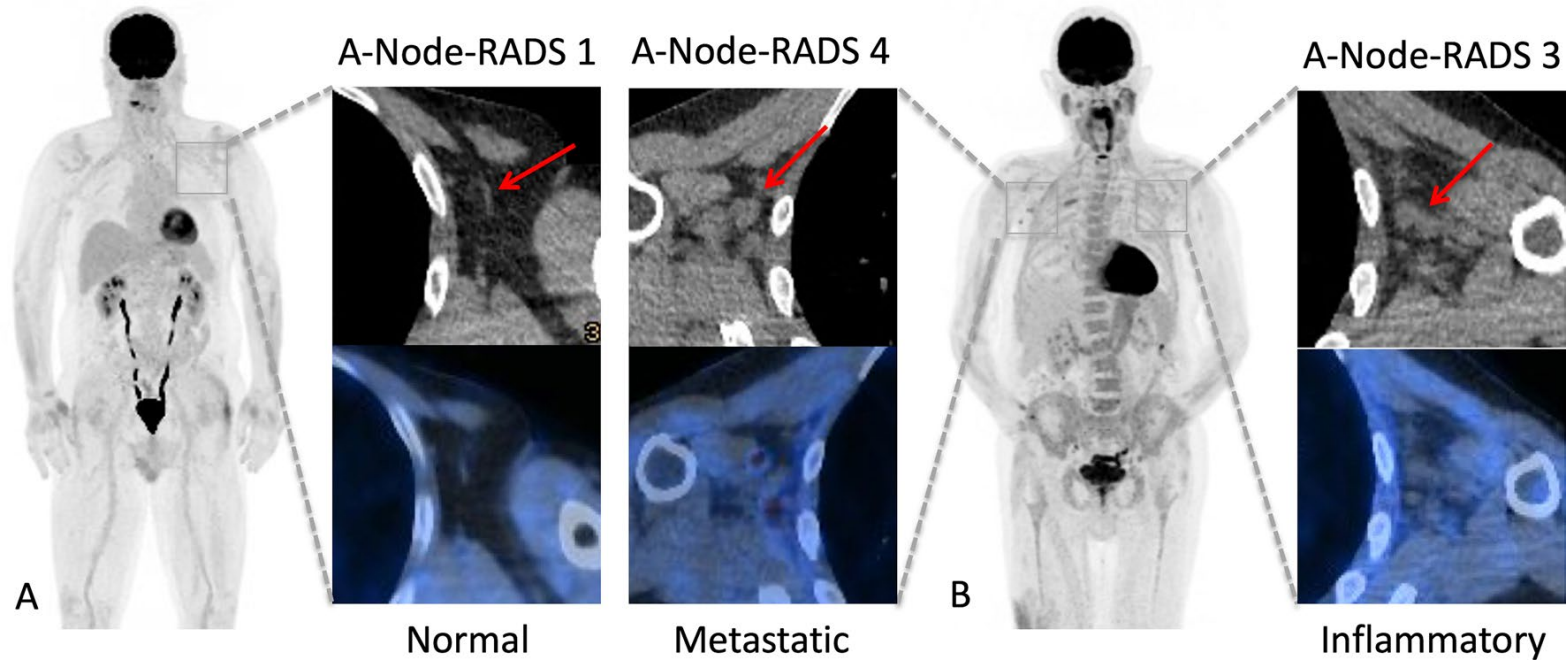
A-Node-RADS evaluation. Adapted Node-RADS (A-Node-RADS) classification of axillary LNs in melanoma patients. An A-Node-RADS 1 LN devoid of metabolic activity was seen in an unvaccinated patient (**A**), whereas active LN graded as A-Node-RADS 3 and 4, characterized as inflammatory and a metastatic LN according to our combined classification, were seen in a vaccinated patient (injection site: left shoulder) who had a melanoma of the cheek (**B**). *A-Node-RADS* adapted version of the node reporting and data system, *LN* lymph node.

HALNs were detected in 42 patients (32.8%), of whom 97.6% were vaccinated. Of those, 2 patients had bilateral HALNs, leading to a total of 44 HALNs evaluated. A per axilla analysis revealed 8 HALNs located in the right axilla (18.2%), whereas the remaining 36 cases were detected in the left axilla (81.8%, *p-*value = 0.001). Based on the combined classification 172 LNs were classified as normal, 30 as inflammatory, and 14 as metastatic. LN characteristics according to patients’ sex, side, and melanoma primary site are reported in Table 2 (Fig. 2). Follow-up data as well as the performances of the combined classification are reported in Supplementary information S1.

**Table 2:** LNs characteristics according to combined classification.

	Normal (n = 172)	Inflammatory (n = 30)	Metastatic (n = 14)	p-value
Sex				
Male	123 (56.9%)	23 (10.6%)	6 (2.8%)	0.11 *X*^*2*^ = 5.7, df = 2
Female	49 (22.7%)	7 (3.3%)	8 (3.7%)	
Side				
Right axilla	100 (46.3%)	3 (1.4%)	5 (2.3%)	< 0.001 *X*^*2*^ = 23.6, df = 2
Left axilla	72 (33.3%)	27 (12.5%)	9 (4.2%)	
Primary melanoma site				
Head	56 (25.9%)	15 (6.9%)	7 (3.2%)	0.12 *X*^*2*^ = 15.5, df = 10
Arms	32 (14.8%)	2 (0.9%)	2 (0.9%)	
Back	38 (17.6%)	3 (1.4%)	1 (0.5%)	
Lower abdomen/genitalia	6 (2.8%)	1 (0.5%)	1 (0.5%)	
Legs	36 (16.7%)	6 (2.8%)	2 (0.9%)	
Unknown	4 (1.8%)	3 (1.4%)	1 (0.5%)	

Data are expressed as absolute values, and absolute percentages.

*LN* lymph node.

### A-Node-RADS

A total of 216 LNs were evaluated on low-dose CT images. A-Node-RADS values are reported in Table 3 (Fig. 2). All 172 LNs classified as normal at combined evaluation were classified as benign by A-Node-RADS (100%), whereas of the 30 inflammatory LNs at combined evaluation, 23 were defined as equivocal (76.7%), 5 (16.7%) as metastatic, and the remaining 2 (6.6%) were characterized as benign. Among metastatic LNs, 6 were deemed to be equivocal (42.9%), while the remaining 8 (57.1%) were defined as metastatic. The relation between the A-Node-RADS and the combined classification is reported in Table 3. The concordance between the two classifications, scored using weighted Cohen’s *k,* was very good (*k*: 0.91, CI 0.86–0.95; *p*-value < 0.001).

**Table 3:** Comparison between A-Node-RADS and combined classification.

	Combined classification	Normal, n = 172	Inflammatory, n = 30	Metastatic, n = 14
A-Node-RADS				
Benign	1	151 (69.9%)	1 (0.5%)	0 (0%)
	2	21 (9.7%)	1 (0.5%)	0 (0%)
Equivocal	3	0 (0%)	23 (10.6%)	6 (2.8%)
Metastatic	4	0 (0%)	5 (2.3%)	7 (3.2%)
	5	0 (0%)	0 (0%)	1 (0.5%)

Data are expressed as absolute values and absolute percentages.

*RADS* reporting and data systems.

### LNs’ A-Node-RADS versus SUV_max_ LNs’ A-Node-RADS in HALNs

In 22/44 HALNs (50.0%) the A-Node-RADS calculated on low-dose CT images correctly identified the LN having the highest SUV_max_ (concordant cases). 63.6% of these were classified as A-Node-RADS 3, whereas 31.8% and 4.5% were classified as 4 and 1, respectively. Table 4 shows the comparison between the A-Node-RADS calculated on low-dose CT images and that of the axillary LN having the highest SUV in discordant cases. The weighted Cohen’s *k* showed good agreement (*k*: 0.64, CI 0.33–0.95; *p*-value < 0.001, Fig. 2).

**Table 4:** Comparison between A-Node-RADS calculated on low-dose CT images and those on the LN with the highest SUV_max_ in patients with HALNs.

A-Node-RADS selected on low-dose CT	Highest LN A-Node-RADS				
	1	2	3	4	5
1	1	0	0	0	0
2	0	0	1	0	0
3	0	0	24	5	0
4	0	0	2	10	0
5	0	0	1	0	0

Data are expressed as absolute values.

*LN* lymph node, *CT* computed tomography, *RADS* reporting and data system, *SUV* standardized uptake value.

### Axillary-to-subcutaneous fat density difference analysis

The median density values measured in the right axilla were lower compared to that of left axilla (median _right axilla_: − 109 HU, IQR: − 114.8 to − 103 HU; median _left axilla_: − 105 HU, IQR: − 112 to − 94.25 HU; *p-*value < 0.001), whereas the median values of subcutaneous tissue did not differ (median _right subcutaneous_: − 120 HU, IQR: − 114 to − 124 HU; median _left subcutaneous_: − 120.5 HU, IQR: − 115 to − 124 HU; *p-*value = 0.96). Similarly, the axillary-to-subcutaneous fat density difference was lower in the right axilla compared to the left axilla (mean _difference right_: 10.4 HU, SD: 8.3 HU; mean _difference left_: 15.5 HU, SD: 11.9 HU; *p-*value < 0.001).

**Figure 3:**
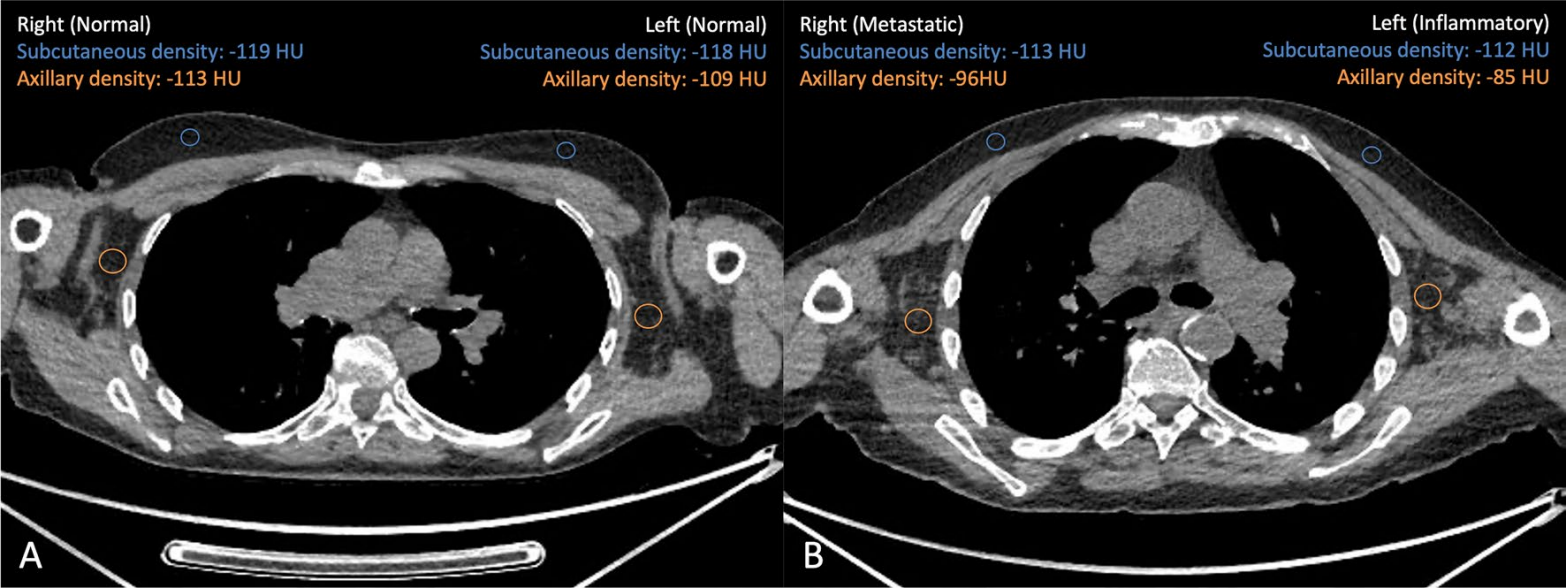
Calculation of the axillary-to-subcutaneous fat density difference. Axillary-to-subcutaneous fat density difference analysis in an unvaccinated patient with a melanoma of the right leg (**A**) and in a vaccinated patient (**B**, injection site: left shoulder) with a melanoma of in the left arm (same patient shown in Supplementary information Fig. S1).

**Figure 4:**
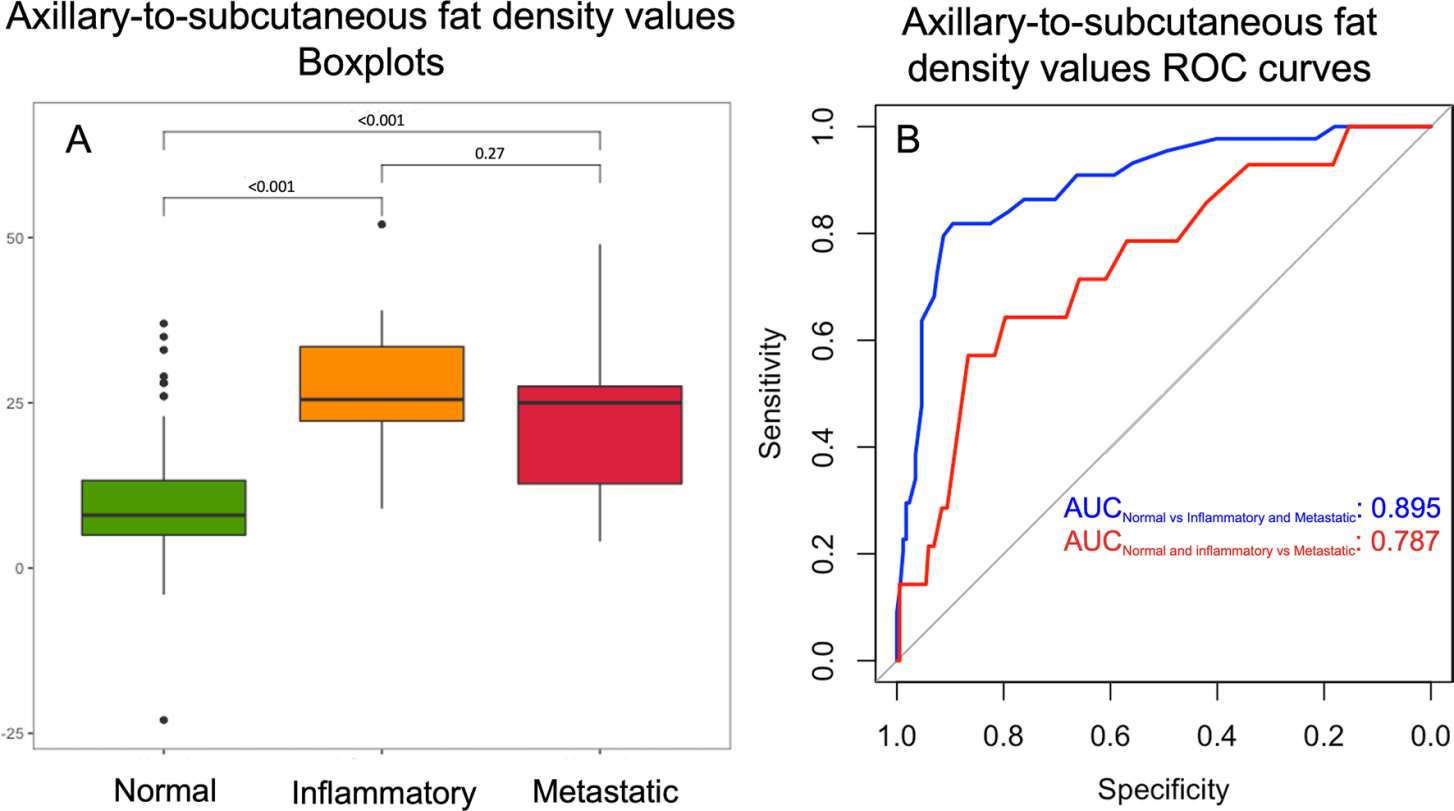
Axillary-to-subcutaneous fat density difference according to the combined classification. Box-plot graph showing the difference values calculated from the axillary-to-subcutaneous fat densities, according to the different categories of the combined classification (**A**). ROC curves with their relative area under the ROC curve, defining the ability of axillary-to-subcutaneous fat density difference to discriminate between normal and inflammatory or metastatic LNs and between normal or inflammatory and metastatic LNs (**B**). *ROC* receiver operating characteristic curve, *LN* lymph node.

Stratifying the values according to the combined classification, the axillary-to-subcutaneous fat density differed between the three groups (*p-*value < 0.001, Figs. 3, 4). Particularly, the values associated with normal LN at combined evaluation were lower than those associated with inflammatory (median _normal_: 8.0 HU, IQR: 5.0–13.8 HU; median _inflammatory_: 25.5 HU, IQR: 22.0–34.3 HU; *p-*value < 0.001) and metastatic ones (median _metastatic_: 25.0 HU, IQR: 11.0–29.0 HU; *p-*value < 0.001). On the contrary, the axillary-to-subcutaneous fat density values did not significantly differ between inflammatory and metastatic LNs (*p-*value = 0.27, Fig. 4). The calculated optimal thresholds to differentiate normal LNs from inflammatory/metastatic ones and normal/inflammatory LNs from metastatic ones using the axillary-to-subcutaneous fat density values, derived by using the Youden index approach, were 20 HU and 21 HU, respectively. These corresponded to the following sensitivity, specificity, and AUC values: 81%, 90%, 0.90, and 64%, 80%, 0.79, respectively (Fig. 4).

## Discussion

Our study showed a high rate of HALNs in patients with melanoma who underwent SARS-CoV-2 vaccination. Interestingly, the A-Node-RADS and the combined classification showed a very good agreement. Furthermore, a good agreement between the A-Node-RADS and A-Node-RADS calculated on the SUV_max_ LN was found. Finally, the axillary-to-subcutaneous fat density difference was lower in normal than in non-normal LNs.

In the recent past, the evidence of both HALNs and enlarged axillary LNs in cancer patients after SARS-CoV-2 vaccination has posed a serious diagnostic dilemma to medical imaging physicians. Of note, a meta-analysis pointed out that HALNs have a higher pooled prevalence than enlarged LNs^15^, highlighting that some HALNs are of normal size. With additional waves of COVID-19 infections striking the westernized countries and booster vaccination doses being administered, the differential diagnosis of HALNs in vaccinated patients remains an unsolved point. Since immunologists agreed that SARS-CoV-2 is likely to become an endemic virus^16^, this diagnostic dilemma is going to become part of the everyday clinical routine of nuclear medicine physicians. As shown in a small cohort study, the anatomical images could ease HALN characterization, reducing equivocal reports^17^. However, thus far, HALN characteristics on CT images have not been standardized, and the HALN interpretation is left to the reader’s experience^12^.

The Node-RADS has been developed to standardize the LN reporting system and to categorize the radiological suspicion, aiming to increase diagnostic performances and serve as a gatekeeper for further exams^14^. However, the benefits of Node-RADS exceeded the expectation proving a high correlation with texture analysis as well^18^. In general, RADS usually place their emphasis on cancer detection^19,20^. However, Node-RADS may help identify low-suspicious HALNs after Sars-CoV-2 vaccination too. Although our results rely on a modified version of the Node-RADS based on low-dose CT images, none of the LNs categorized as metastatic at combined classification had a benign aspect (A-Node-RADS 1 or 2), and none of those devoid of 2-[^18^F]-FDG activity had equivocal or highly suspicious characteristics (A-Node-RADS 3 to 5), proving the potential of A-Node-RADS as a reliable tool to differentiate benign from malignant LNs. Moreover, 83.3% of the inflammatory LNs did not have a clearly malignant aspect (A-Node-RADS 1 to 3), stressing the usefulness of anatomical imaging in characterizing HALNs. Also, 57.1% of metastatic LNs were correctly classified as highly or very highly suspicious LNs by A-Node-RADS (grade 4 or 5), while the remaining 42.9% were deemed to be equivocal (grade 3). These performances could relate to the high correlation between LN size and shape and texture features, which in turn relate to LN and tissue microstructure^18^.

Interestingly, most inflammatory LNs at combined classification (76.7%) had an equivocal aspect at A-Node-RADS (grade 3). Although the histological evaluation shows clear differences between reactive and metastatic LNs^21,22^, this distinction in images remains challenging^23^. Indeed, inflammatory and metastatic LN could share imaging features such as 2-[^18^F]-FDG uptake, dimensional enlargement, and cortical thickening. Also, it is worth mentioning that most of the imaging studies investigating LN characterization based their assumptions on a dichotomous paradigm (benign vs. malignant or inflammatory vs. malignant)^24,25^, while in our study, three scenarios have been evaluated.

The correct interpretation of equivocal cases (RADS grade 3) is also problematic in other organs^26–29^. In fact, Elsholtz et al*.* specified that the metastatic risk in a patient with an LN characterized as Node-RADS 3, should be weighted considering the stage and histological grade of the primary tumor^14^. This is in line with the results of a recent study showing only 50% of LNs graded as Node-RADS 3 and 43% of those graded 4 were positive at pathological analysis, in patients with bladder cancer^30^. Similarly, studies on other organs highlighted that the probability of harboring a tumor in a grade 3 RADS is relatively low^26–29^. Additionally, none of the patients who underwent fine needle aspiration due to enlarged axillary LNs after SARS-CoV-2 vaccination showed malignant cells^31^. Therefore, the majority of HALNs in vaccinated patients are unlikely to represent a metastasis at subjective evaluation, irrespective of the primary tumor^9^. Although our study is based on A-Node-RADS rather than on Node-RADS, we believe that A-Node-RADS 3 has the potential to become a useful tool to diagnose inflammatory LN after SARS-CoV-2 vaccination. However, further studies specifically testing this hypothesis are needed.

As shown by our results, the axillary-to-subcutaneous fat density difference may be a simple yet valid tool to distinguish between benign and non-benign LNs. Indeed, inflammation and tumor dissemination induce an increase in lymphatic vessel number, caliber, and permeability^22,32^, as well as perinodal exudate^33^. These conditions may explain the results of our study. Indeed, perivisceral fat analysis already proved its usefulness in other organs, such as the vascular system, rectum, or mesentery^34–37^, but, to the best of our knowledge, this is the first study analyzing its ability to categorize axillary LNs. Of note, while our study showed good diagnostic accuracy in differentiating between normal and inflammatory or metastatic LNs, it was not able to discriminate between inflammatory and metastatic LNs. Nonetheless, our preliminary results strongly motivate further analysis of this promising application of unenhanced CT images.

Our study has several limitations. First, it is a retrospective, single-center evaluation with all the inherent limitations of this study design. Second, the study lacks a correlation between the combined PET classification and the histological analysis thus it is prone to misclassifications. Relying on 2-[^18^F]-FDG findings to classify LNs could have been misleading, opening to both false positives (ex. unknown inflammatory processes, such as hidradenitis) and false negatives (ex. micromestastasis in axillary LNs). However, most of the papers dealing with HALNs after Sars-CoV-2 vaccination share the same limitation since the rate of histological confirmation is low. Although we tried to overcome some of these drawbacks by providing follow-up data, the latter were not available for all our patients. Hence, we opted to rely on an ad-hoc classification, which proved to be highly accurate but had low sensitivity values. Third, the A-Node-RADS used in this paper slightly differs from the original classification, because of the absence of contrast medium administration. Although the Author of the Node-RADS stated that the use of contrast medium is mandatory to properly classify them^14^ the conservative approach used in our study to define LN’s necrosis yielded a good correlation with the combined classification. Finally, our results cannot be generalized to other vaccines because of the different immune reactogenicity.

## Conclusions

A-Node-RADS and axillary-to-subcutaneous fat density difference can support clinical decisions, helping nuclear medicine physicians discriminate between normal, inflammatory, and metastatic LNs in patients with melanoma who underwent 2-[^18^F]-FDG PET/CT after SARS-CoV-2 vaccination.

## Methods

In this retrospective, cross-sectional, single-center study, patients with proven melanoma who underwent a 2-[^18^F]-FDG PET/CT at the Nuclear Medicine Department of the University Hospital of Zurich between the 2nd of February and the 6th of April 2021 were analyzed. Clinical data, such as vaccination status, either with BNT162b2 (Comirnaty®, Pfizer/BioNTech, New York, USA/Mainz, Germany) or mRNA-1273 (Moderna®, Moderna Biotech, Cambridge, USA), vaccination dates, age, sex, body mass index (BMI), primary melanoma site were derived from clinical records or by telephone interviews. Follow-up data were based on 2-[^18^F]-FDG PET/CT images and reports. The inclusion criteria were: (1) age > 18 years old, (2) diagnosis of melanoma, and (3) having undergone a 2-[^18^F]-FDG PET/CT. The exclusion criteria were: (1) inadequate quality of low-dose CT images, (2) previous metastatic involvement of axillary LNs, defined as HALNs detected in the penultimate 2-[^18^F]-FDG PET/CT exam, (3) absence of subcutaneous tissue in the axillary or pectoral region, and (4) presence of concomitant tumors.

Melanoma’s primary sites were categorized into the following groups: head, left arm, right arm, back, lower abdomen/genitalia, leg, and unknown. The “head” category included melanomas arising between the calvarium and the inferior part of the neck, defined as a plane passing through the superior margin of clavicles and the superior edge of the trapezius muscle. The group “arms” included all the melanomas involving the scapula and pectoral regions, arm, forearm, and hand, whereas the “back” gathered tumors involving the remaining portions of the back. Finally, the “unknown” category grouped patients who had melanoma, but clinical information did not specify the primary melanoma site.

The Swiss Association of Research Ethics Committees approved this study (Trial No. 2021-00444), which was conducted according to ICH-Good Clinical Practice rules and the Declaration of Helsinki. Written informed consent was obtained from all patients.

### Image acquisition

Each patient was imaged using a last-generation PET/CT scanner (GE Discovery MI, GE Healthcare, Waukesha, WI). The exam protocol consisted of a low-dose, attenuation correction, spiral CT scan (collimation width: 0.625 mm, pitch: 0.98, kVp: 120, automatic tube dose modulation ranging between 15 and 100 mAs, matrix: 512 × 512, slice thickness: 1.25 mm, spacing between slices: 1.25 mm), displayed using body filter (W/L: 40/400 Hounsfield Unit, HU), and a 2-[^18^F]-FDG PET scan. The scan length did not vary between CT and PET exams, usually extending between the calvarium and mid-thighs. In selected cases, it was prolonged up to the feet. PET images were acquired after a minimum of 4 h fasting before the 2-[^18^F]-FDG injection. 2-[^18^F]-FDG uptake time was set to 60 min. The PET acquisition time was 2 min per bed position; patients’ bed positions differed according to patient size, ranging between 6 and 11, with a 23% overlap (17 slices). PET reconstructions were generated using penalized likelihood reconstruction (Q. Clear, GE Healthcare) with a *β*-value of 450, with a 256 × 256 matrix.

### CT image analysis

A board-certified radiologist with 7 years of experience in oncologic imaging (A.G.G.), blinded to both vaccination status and PET results, reviewed the low-dose CT images. The left and right axilla’s LNs were analyzed using a four-step approach. First, the radiologist defined the target LN in each axilla. Second, axial, coronal, and sagittal reconstructions were evaluated, selecting the best one to image the LN; oblique reconstructions were generated if needed. Third, LN’s short and long diameters, cortical thickness, presence of fat hilum, and/or cortical lump were recorded. Subsequently, a modified version of the Node-RADS, the A-Node-RADS, was calculated^14^ as presented in Table 5. Of note, the original version of the Node-RADS applies to post-contrast images to characterize the texture of the LN, whereas our study relied on unenhanced low-dose CT images only. Therefore, we adopted a dimensional approach to characterize this parameter. Since Don et al*.*, Zoumalan et al*.*, and Yang W.T. et al*.* proved necrosis to be directly related to LN’s short axis we decided to arbitrarily generate a dimensional cutoff for LN necrosis based on their studies^38–40^. An axial diameter of 18 mm was selected as the cutoff for necrosis, which was calculated by averaging the cutoffs proposed in the aforementioned studies (20, 13, and 23 mm, respectively). To account for focal necrosis, an LN cortex thickness of > 6 mm was arbitrarily selected as necrosis cutoff, based on Grimm et al*.*, who showed that less than 95% of normal axillary LN have a cortex thickness > 6 mm^41^. These parameters were combined with fatty hilum presence to define the four distinct texture subcategories proposed in the Node-RADS. Finally, a screenshot of the selected LN was taken for further comparison.

**Table 5:** Adapted Node-RADS classification.

Criteria	Anatomical characteristics	Definition	Points
Size	Short LN diameter ≤ 10 mm	Normal	0
	Short LN diameter between 11 and 30 mm	Enlarged	1
	Short LN diameter > 30 mm	Bulk	5
Shape	Detectable fatty hilum and a short-to-long LN’s diameters ratio < 0.9	Bean shaped LN with detectable fatty hilum	0
	Undetectable fatty hilum and a short-to-long LN’s diameters ratio ≥ 0.9	Spherical without fatty hilum	1
Border	No cortical lump on the LN cortex	Smooth borders	0
	Presence of cortical lump on the LN’s cortex	Irregular borders	1
Texture	Detectable fatty hilum, a LN’s short diameter < 9 mm and cortex thickness < 6 mm	Homogeneous	0
	Detectable fatty hilum, a LN’s short diameter between 10 and 18 mm and a cortex thickness < 6 mm	Heterogeneous	1
	Detectable fatty hilum, but a LN’s short diameter between 10 and 18 mm, and a LN’s cortex thickness > 6 mm	Focal necrosis	2
	Non-detectable fatty hilum and a short diameter ≥ 18 mm	Gross necrosis or any other new necrosis	3

*RADS* reporting and data system, *LN* lymph node.

After A-Node-RADS calculation, circular regions of interest (ROIs) were drawn in the axillary fat (average diameter: 10 mm) and in the pectoral subcutaneous tissue (average diameter: 5 mm) of each side. Lateral thoracic and subscapular vessels were carefully avoided while drawing the ROIs in the axillary fat. The axillary-to-subcutaneous fat density difference was subsequently calculated aiming to minimize image noise.

### PET image analysis: qualitative and quantitative assessment

Two weeks after the CT reading session, the same reader inspected the three-dimensional maximum intensity projection (MIP) 2-[^18^F]-FDG PET images of each patient distinguishing positive (evidence of HALN) from negative cases. According to previous literature, HALNs were defined as LN that were visually depictable on MIP images^9^. In positive cases, a semi-automated cubicle volume of interest encasing the LN with the highest metabolic activity was drawn on fused PET/CT axial images. Metabolic activity was based on the maximum standardized uptake value (SUV_max_), which was calculated as the decay corrected radioactivity per volume (kBq/ml), divided by the initially injected dose (MBq) and multiplied by body weight (kg). In positive cases, the stored CT screenshots were compared to PET images. Concordant cases were those in which the same LN was pinpointed by both techniques, while discordant cases were those in which the LNs differed (qualitative analysis). In discordant cases, the A-Node-RADS criteria were calculated subsequently on the LN having the highest SUV_max_ (quantitative approach).

### Combined classification quantifying the metastatic risk score

The risk of LN metastatic involvement was weighted according to the following clinical and imaging parameters: LN’s metabolic activity, primary melanoma site, vaccination status, and metastasis in other organs (combined classification). These parameters were used to impute a quantitative scale grading the risk of metastatic involvement of the LN. Each LN was categorized as inactive (0 points) or active according to 2-[^18^F]-FDG uptake at MIP images. HALNs concordant to the primary melanoma site were further divided into ipsilateral (1 point) or contralateral to the vaccination site (1.5 points). Similarly, these categories were used to describe active LN discordant to the primary melanoma site but with a different weighting (0.5 points and 1.5 points, respectively). As suggested in Sollini et al*.*^42^, different weights were used to grade the LN metastatic risk according to the primary melanoma site. Moreover, the metastatic involvement of other organs was also counted (1 point). Points were summed, and the following categories were created: normal = 0 points, inflammatory 0 < x ≤ 1, metastatic > 1 point (Supplementary information Fig. S1).

The combined classification was used as the reference standard; both the Node-RADS and the axillary-to-subcutaneous fat density changes were compared to it.

In a subset of patients, follow-up data were available, and the performances of the combined classification were explored. Metastatic LNs were defined as those showing a (1) de-novo hypermetabolic pattern or those increasing their metabolism at follow-up exam and (2) reported as metastatic. In patients who reported further rounds of vaccination, hypermetabolic LNs ipsilateral to the injection site were deemed normal. LNs devoid of metabolic activity were defined as normal. Positive LNs at combined classification were those categorized as metastatic at follow-up, while those categorized as negative or inflammatory were defined as negative.

### Statistical analysis

Statistical analysis was performed using R (version 4.0.5, https://www.r-project.org). D’Agostino-Pearson test was used to check normality. Continuous variables were presented as mean ± standard deviation (SD) and compared using the analysis of variance (ANOVA) or unpaired Student *t-* test. Not normally distributed variables were presented as median with interquartile range (IQR) and compared using the Kruskal–Wallis or the Mann–Whitney test. Finally, categorical variables were presented as frequencies and percentages and compared using Fisher’s exact test or Chi-square test.

A-Node-RADS was grouped into the following categories: benign LNs (gathering A-Node-RADS 1 and 2), equivocal LNs (A-Node-RADS 3), and malignant LNs (gathering A-Node-RADS 4 and 5). Fleiss-Cohen weighted (quadratic) Cohen’s kappa (*k*) was used to evaluate the agreement between the A-Node-RADS and the combined classification, as well as between the A-Node-RADS value of the LN selected on CT images and the SUV_max_ LN^43^. *k* value agreement was graded as follows: poor (*k* value < 0.20), fair (≥ 0.20 and < 0.40), moderate (≥ 0.40 and < 0.60), good (≥ 0.60 and < 0.80), and very good (≥ 0.80 up to 1). The *k* values were presented together with a 95% confidence interval (CI) calculated using Wald’s method.

The axillary-to-subcutaneous fat density differences were compared according to the combined classification. The Youden index was used to define the best sensitivity and specificity cut-offs for the axillary-to-subcutaneous fat density in discriminating between normal and inflammatory/metastatic LNs as well as between normal/inflammatory and metastatic LNs. Moreover, the area under the receiver operating characteristic curve was calculated (ROC, AUC).

The sensitivity, specificity, and accuracy values of the combined classification according to follow-up data were presented together with 95% CI.

For all tests, a *p-*value < 0.05 was considered to indicate a statistically significant difference. The Holm correction was used in the case of multiple comparisons, keeping the statistical significance set at 0.05.

### Ethics approval

The present study was approved by the Swiss Association of Research Ethics Committees and was conducted in compliance with ICH-GCP rules and the Declaration of Helsinki. Trial No. 2021-00444.

### Informed consent

Only patients with documented willingness to the use of their medical data for research were included.

### Supplementary Information

Supplementary Information.

## Data Availability

The datasets analyzed in the current study are not publicly available due to the possibility to showcase patients’ identity. However, they could be available from the corresponding author upon reasonable request.
