# Supplementary material for: Characterization of hypermetabolic lymph nodes after SARS-CoV-2 vaccination using PET-CT derived node-RADS, in patients with melanoma
Source: Sci Rep. 2023 Oct 26;13:18357. doi: 10.1038/s41598-023-44215-2 (PMC10603100; doi:10.1038/s41598-023-44215-2)
Supplement: Supplementary file 1 — Supplementary Information. [file 41598_2023_44215_MOESM1_ESM.pdf]

Characterization of Hypermetabolic Lymph Nodes After SARS-CoV-2 Vaccination Using PET-CT Derived Node-RADS, in Patients with Melanoma

Antonio G. **Gennari**, MD, Alexia **Rossi**, MD, PhD, Thomas **Sartoretti**, BSc, Alexander **Maurer**, MD, Stephan **Skawran**, MD, Valerie **Treyer**, PhD, Elisabeth **Sartoretti**, MD, Alessandra **Curioni-Fontecedro**, MD, Moritz **Schwyzer**, MD, Stephan **Waelti**, Martin **Huellner**, MD, Michael **Messerli**, MD

Supplementary material - Follow-up information and performances of the combined classification

Follow-up data were available for 98 of 108 patients (196 LNs), with a median follow-up of 4 months (IQR: 3 to 6 months). Results were as follows:

		Categorization based on follow-up information	
		Normal	Metastatic
Categorization based on combined classification	Normal	174	9
	Metastatic	10	3

Therefore, the specificity, sensitivity, and accuracy of the combined classification used as the gold standard in this study were 95% (CI: 90 to 97%), 25% (CI: 6 to 57%), and 90% (CI: 85 to 94%), respectively.
In those who did not have follow-up data available, a single LN was graded as metastatic, 2 were graded as inflammatory, and the remaining 17 were graded as normal.

Supplementary Figure – Flow diagram describing the combined classification and an illustrative case of a vaccinated patient (injection site: left shoulder) who previously had a melanoma in the left arm. 2-[^{18}F]-FDG: 2-[^{18}F]-fluoro-2-deoxy-D-glucose

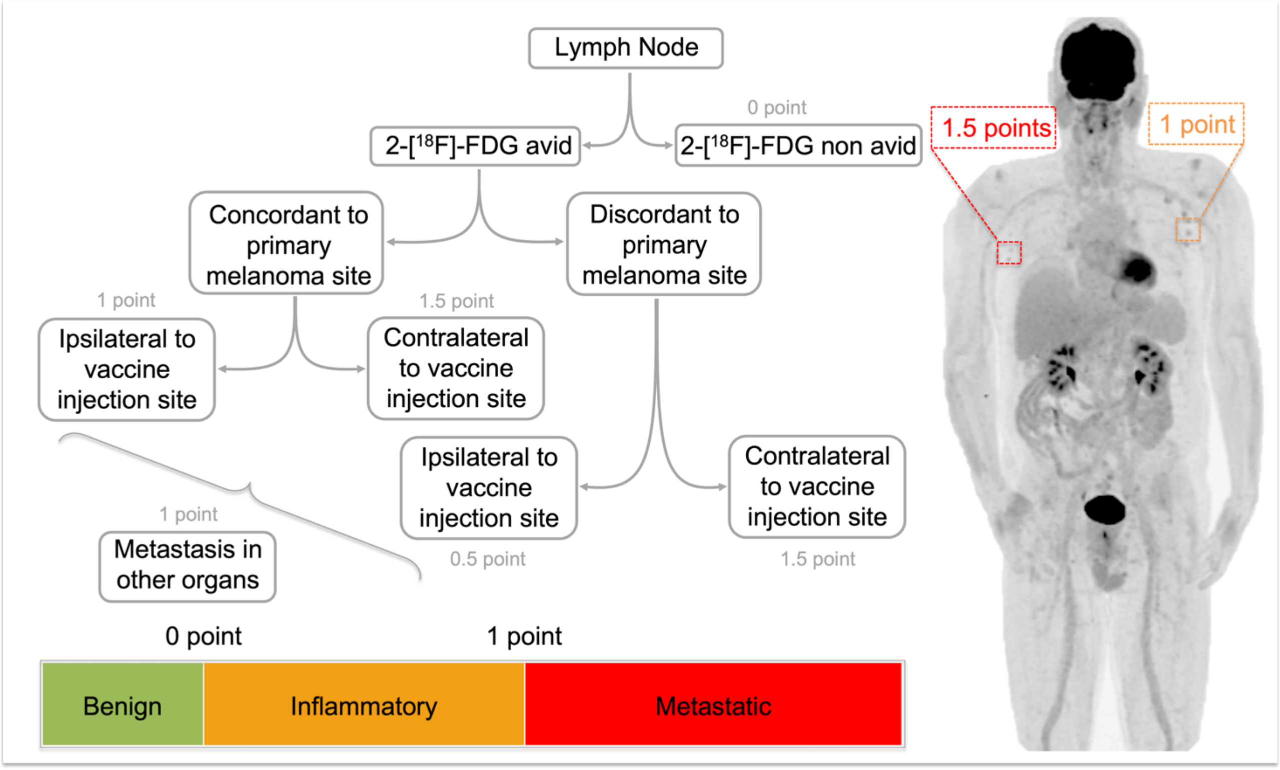

2-[^{18}F]-FDG: 2-[^{18}F]-fluoro-2-deoxy-D-glucose
